# Bioequivalence study of two formulations of a sitagliptin/metformin hydrochloride extended-release tablet in healthy Chinese subjects under fasting and fed conditions: a randomized, open-label, single-dose, crossover trial

**DOI:** 10.3389/fphar.2026.1927547

**Published:** 2026-10-14

**Authors:** Lin Fang, Xin Li, Rongxin Ban, Chenjing Wang, Yi Xu, Lipeng Xue, Ping Shi, Feifei Sun, Yu Cao

**Affiliations:** 1 Phase I Clinical Research Center, The Affiliated Hospital of Qingdao University, Qingdao, China; 2 Clinical Research Department, Qilu Pharmaceutical (Hainan) Co., Ltd., Haikou, Hainan, China

**Keywords:** bioequivalence, extended-release tablet, metformin, pharmacokinetics, sitagliptin

## Abstract

**Introduction:**

This study aimed to evaluate the bioequivalence and safety of a newly developed generic sitagliptin/metformin hydrochloride extended‐release (XR) tablet (50 mg/500 mg) compared with the reference formulation, Janumet XR, in healthy Chinese subjects under fasting and fed conditions.

**Methods:**

This randomized, open‐label, single‐dose, two‐period, two‐sequence crossover study included 40 subjects in the fasting cohort and 36 subjects in the fed cohort. Subjects received a single dose of the test or reference formulation in each period, separated by a 5‐day washout period. Plasma concentrations of sitagliptin and metformin were determined using a validated liquid chromatography‐tandem mass spectrometry method. Bioequivalence was assessed using the 90% confidence intervals (CIs) for the geometric mean ratios (GMRs; test/reference) of maximum plasma concentration (C_max_), area under the plasma concentration‐time curve from time zero to the last quantifiable concentration (AUC_0‐t_), and area under the plasma concentration‐time curve from time zero to infinity (AUC_0‐infinity_).

**Results:**

Under fasting conditions, the 90% CIs for the GMRs of sitagliptin for C_max_, AUC_0‐t_, and AUC_0‐infinity_ were 88.24%–101.46%, 98.24%–103.15%, and 98.41%–103.33%, respectively. The corresponding values for metformin were 93.23%–103.26%, 93.00%–106.20%, and 92.89%–105.74%. Bioequivalence was also established under fed conditions for sitagliptin (C_max_: 88.29%–99.06%; AUC_0‐t_: 96.19%–101.02%; AUC_0‐infinity_: 96.07%–101.04%) and metformin (C_max_: 92.06% –99.39%; AUC_0‐t_: 91.31%–100.06%; AUC_0‐infinity_: 91.00%–99.30%). Analysis of variance showed a significant period effect for metformin AUC_0‐t_ and AUC_0‐infinity_ under fasting conditions (P < 0.05), whereas no significant formulation effect was observed (P > 0.05). Both formulations were well tolerated. Only Grade 1 or Grade 2 adverse events were reported, and no serious adverse events occurred

**Discussion:**

The generic sitagliptin/metformin hydrochloride XR tablet was bioequivalent to the reference product under both fasting and fed conditions in healthy Chinese subjects. The generic formulation was well tolerated and may provide a safe and effective alternative for the treatment of type 2 diabetes mellitus.

## 1 Introduction

China bears one of the largest diabetes burdens worldwide, and type 2 diabetes mellitus (T2DM) remains a major public health challenge (Jin et al., 2024; Zheng et al., 2018). Sustained glycemic control is essential for reducing the risk of diabetes-related complications, yet monotherapy is often insufficient to maintain glycemic targets over time (Davies et al., 2022; Turner et al., 1999). Accordingly, combination therapy with agents that have complementary mechanisms of action is widely used in the management of T2DM (Davies et al., 2022; Blonde et al., 2014).

Sitagliptin, a dipeptidyl peptidase-4 (DPP-4) inhibitor, enhances glucose-dependent insulin secretion and suppresses glucagon release (Gallwitz, 2019), whereas metformin improves insulin sensitivity and reduces hepatic glucose production (Foretz et al., 2019). The fixed-dose combination of sitagliptin and metformin therefore represents a rational therapeutic option for T2DM (Blonde et al., 2014; Goldstein et al., 2007). In addition, the metformin hydrochloride extended-release component allows once-daily administration and has been associated with a lower incidence of dyspepsia than immediate-release metformin (Schwartz et al., 2006; Abrilla et al., 2021).

Janumet® XR (sitagliptin/metformin hydrochloride extended-release tablets), intended for once-daily administration with a meal, is an established reference product (Sharp and Dohme, 2020), and the development of high-quality generic alternatives is crucial for improving drug accessibility and reducing long-term treatment costs. Therefore, the present study aimed to assess the pharmacokinetics, bioequivalence, and safety of a generic sitagliptin/metformin hydrochloride extended-release tablet (50 mg/500 mg) compared with the reference formulation in healthy Chinese subjects under both fasting and fed conditions.

## 2 Materials and methods

### 2.1 Study design

This was an open-label, randomized, single-dose, two-period, two-sequence crossover bioequivalence study conducted in healthy Chinese subjects under fasting and fed conditions, which is consistent with standard bioequivalence study design principles. Subjects in the fasting and fed cohorts were randomized separately. In each cohort, subjects were assigned in a 1:1 ratio to one of two treatment sequences (T–R or R–T), with a 5-day washout period between dosing periods. The test formulation was sitagliptin/metformin hydrochloride extended-release tablets (50 mg/500 mg; batch No. KCIL2003), manufactured by Qilu Pharmaceutical (Hainan) Co., Ltd. The reference formulation was Janumet* XR 50 mg/500 mg (sitagliptin/metformin hydrochloride extended-release tablets; batch No. U029591), manufactured by Merck Sharp & Dohme Corp.

In the fasting cohort, subjects received a single oral dose of the test or reference formulation with 240 mL of warm water after an overnight fast of at least 10 h. In the fed cohort, subjects consumed a high-fat, high-calorie meal providing approximately 900 kcal within 30 min before dosing. The meal provided 14.7% of calories from protein, 55.7% from fat, and 29.6% from carbohydrates. In both cohorts, 60 mL of 20% glucose solution plus 30 mL of warm water was administered at 1 and 2 h post-dose. Water intake was prohibited within 1 h before and after dosing, and a standardized lunch was provided 4 h after dosing. The study drug was swallowed whole without chewing, crushing, or splitting.

The sample size was determined in accordance with the Technical Guidelines for Human Bioequivalence Studies with Pharmacokinetic Endpoints for Chemical Generic Drugs issued by the National Medical Products Administration (NMPA) of China. Based on pharmacokinetic data from the originator’s pharmacokinetic bridging study (Study P147) (U.S. Food and Drug Administration and Center for Drug Evaluation and Research, 2012), sitagliptin/metformin hydrochloride extended-release tablets were considered unlikely to be highly variable drugs. Assuming a true geometric mean ratio of 0.90, an intra-subject coefficient of variation of 20%, a significance level of 0.05 for the two one-sided tests (TOST), and 80% power, 37 subjects were required to demonstrate bioequivalence under fasting conditions. To allow for potential dropouts and other uncertainties, 40 subjects were planned for enrollment in the fasting cohort. For the fed cohort, based on previous studies of sitagliptin phosphate monotherapy, intra-subject variability under fed conditions was anticipated to be lower than that under fasting conditions; therefore, 36 subjects were planned for enrollment. In total, 76 subjects were planned for enrollment, including 40 in the fasting cohort and 36 in the fed cohort.

The protocol was pre-approved by the Medical Ethics Committee of the Affiliated Hospital of Qingdao University on 9 November 2022 (approval No. QYFYEC2022-133-01). Written informed consent was obtained from all subjects before screening. The trial was prospectively registered on the National Medical Products Administration (NMPA) Registration and Information Disclosure Platform for Drug Clinical Studies (http://www.chinadrugtrials.org.cn, identifier: CTR20223071). Although this mandatory official registry for regulatory drug approval in China currently lacks formal recognition by the World Health Organization (WHO), to strictly comply with the publication requirements of the International Committee of Medical Journal Editors (ICMJE), this bioequivalence trial underwent retrospective registration with ClinicalTrials.gov, a WHO-accredited primary registry (https://clinicaltrials.gov/) (identifier: NCT07679711). The study was conducted in accordance with the Declaration of Helsinki, the Good Clinical Practice guidelines issued by the NMPA, and applicable local regulations.

### 2.2 Subjects

Eligible participants were healthy male or female adults aged 18 years or older, with no prespecified upper age limit, a body weight of at least 50.0 kg for males and 45.0 kg for females, and a body mass index (BMI) of 19.0–27.0 kg/m^2^. Screening assessments included a review of medical history, physical examination, vital signs, 12-lead electrocardiogram (ECG), clinical laboratory testing, serological testing, urine drug screening, and pregnancy testing for female subjects.

Key exclusion criteria included clinically significant abnormalities identified during screening; a history of clinically significant allergy, pancreatitis, or hypoglycemia; an estimated glomerular filtration rate (eGFR) < 90 mL/min/1.73 m^2^; gastrointestinal disorders that might affect drug absorption; positive tests for hepatitis B surface antigen, hepatitis C antibody, human immunodeficiency virus (HIV) antibody, or syphilis antibody; drug abuse, excessive alcohol consumption, or heavy smoking; recent surgery, blood donation, or participation in another clinical trial; and the use of prescription drugs, over-the-counter drugs, herbal medicines, or health products within the protocol-defined restricted periods. Detailed inclusion and exclusion criteria are provided in Supplementary Appendix S1.

### 2.3 Blood sampling and bioanalytical method

In both the fasting and fed cohorts, venous blood samples (approximately 4 mL each) were collected into K2EDTA tubes at pre-dose (within 60 min prior to dosing) and at 0.33, 0.67, 1.0, 1.33, 1.67, 2.0, 2.33, 2.67, 3.0, 3.5, 4.0, 4.5, 5.0, 6.0, 7.0, 8.0, 10.0, 12.0, 24.0, 32.0, and 48.0 h post-dose. When an indwelling catheter was used, approximately 1 mL of blood was discarded before each collection.

The collected blood samples were centrifuged within 1 h after collection at 1700 × g for 10 min at approximately 4 °C (2 °C–8 °C). The separated plasma was divided into two aliquots, one for analysis and one as a backup, and stored below −60 °C until analysis. Plasma concentrations of sitagliptin and metformin were determined using a validated liquid chromatography–tandem mass spectrometry (LC–MS/MS) method. The calibration range was 1.00–500 ng/mL for sitagliptin, with a lower limit of quantification (LLOQ) of 1.00 ng/mL, and 8.00–4000 ng/mL for metformin, with an LLOQ of 8.00 ng/mL. Sample handling, storage, and bioanalysis were performed in accordance with the study protocol, relevant NMPA guidelines, and the bioanalytical laboratory’s standard operating procedures.

### 2.4 Pharmacokinetic and statistical analysis

Pharmacokinetic parameters were calculated by noncompartmental analysis using Phoenix WinNonlin version 8.2 (Certara, Princeton, NJ, USA). The primary pharmacokinetic parameters for both sitagliptin and metformin were maximum plasma concentration (C_max_), area under the plasma concentration–time curve from time zero to the last quantifiable concentration (AUC_0–t_), and area under the plasma concentration–time curve from time zero to infinity ( ${\text{AUC}}_{0–\infty }$ ); the secondary parameters were time to reach C_max_ (T_max_), terminal elimination half-life (t_1/2_), and terminal elimination rate constant (λ_z_).

Bioequivalence was assessed using the natural logarithm-transformed primary pharmacokinetic parameters. An analysis of variance (ANOVA) model was applied with sequence, period, and formulation as fixed effects and subject nested within sequence as a random effect. Geometric mean ratios (GMRs; test/reference) and their 90% confidence intervals (CIs) were calculated for C_max_, AUC_0–t_, and ${\text{AUC}}_{0–\infty }$ . Bioequivalence was established if the 90% confidence intervals for the geometric mean ratios of all primary pharmacokinetic parameters of both sitagliptin and metformin were within 80.00%–125.00%. Tmax was compared using the Wilcoxon signed-rank test. Descriptive statistics of pharmacokinetic parameters and concentration–time data were generated using Phoenix WinNonlin version 8.2, and nonparametric analysis of Tmax, demographic characteristics, and safety analyses were performed using SAS version 9.4 (SAS Institute Inc., Cary, NC, USA).

### 2.5 Safety assessment

Safety was evaluated by monitoring adverse events (AEs), serious adverse events (SAEs), vital signs, physical examinations, clinical laboratory tests, and 12-lead ECGs. Vital signs, including body temperature, pulse rate, and blood pressure, were measured at pre-dose (within 60 min prior to dosing) and at 1, 3, 8, 12, 24, and 48 h post-dose in each period. AEs were collected throughout the study by spontaneous reporting and non-leading questioning, and subjects with AEs were followed until resolution, stabilization, loss to follow-up, or refusal of further follow-up. The severity of AEs was graded according to the National Cancer Institute Common Terminology Criteria for Adverse Events (NCI CTCAE), version 5.0.

## 3 Results

### 3.1 Study population

A total of 160 healthy Chinese adult volunteers were screened between 14 February 2023, and 22 March 2023. Ultimately, 40 subjects were included in the fasting cohort and 36 subjects were included in the fed cohort. In the fasting cohort, three subjects discontinued the trial: two withdrew prior to Period two dosing (one due to an AE of elevated blood human chorionic gonadotropin levels, and one for personal reasons), and one withdrew after completing Period two dosing for personal reasons. The remaining 37 subjects completed both periods. In the fed cohort, one subject was withdrawn prior to dosing in Period one due to failure to consume the high-fat, high-calorie meal within the required timeframe. Another subject withdrew following an epileptic seizure post-dosing in Period 1 (later confirmed to have been concealed during screening). Ultimately, 34 subjects completed both periods (Figure 1).

**FIGURE 1:**
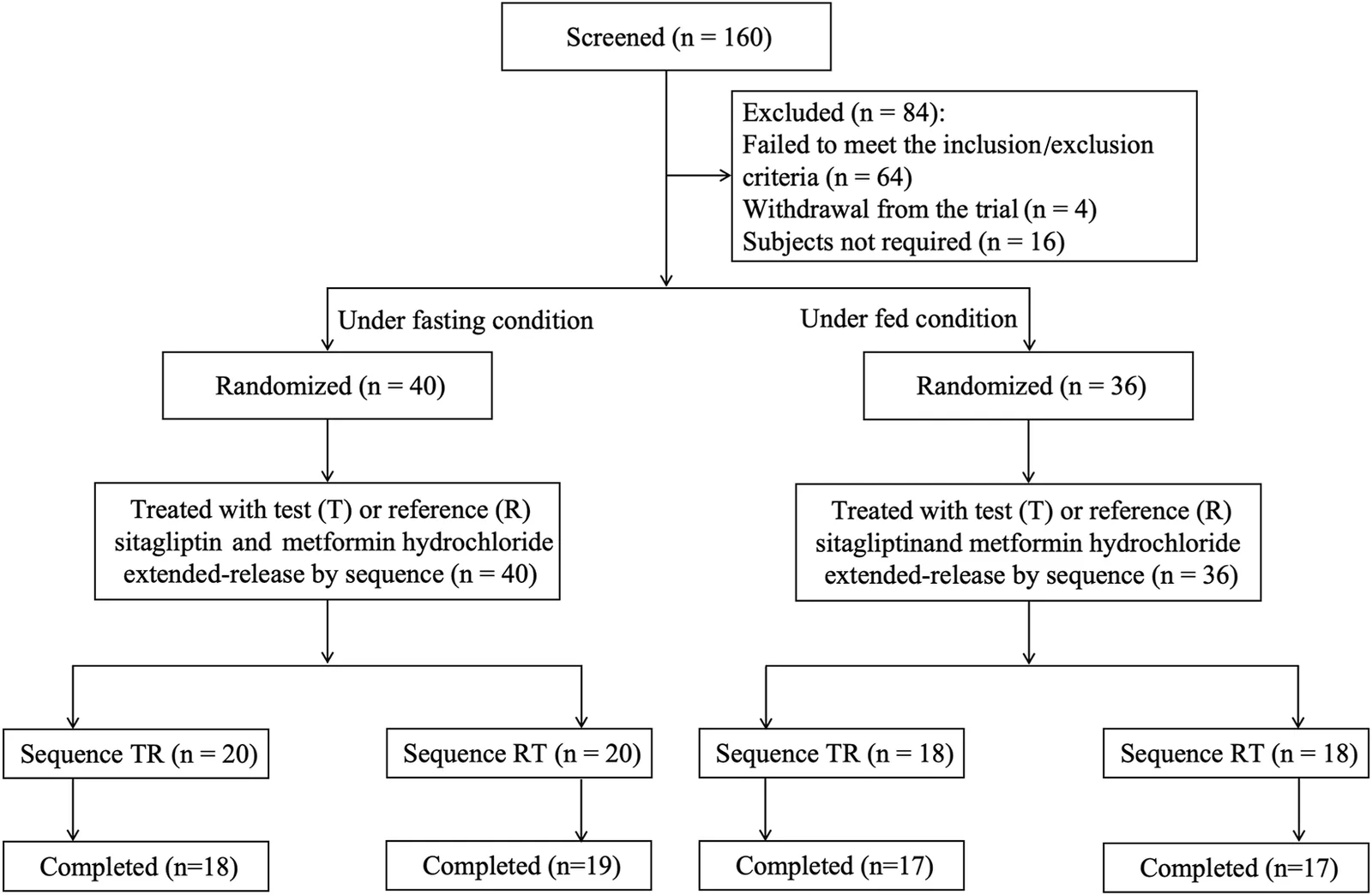
CONSORT flow diagram of subject disposition. T, test formulation; R, reference formulation.

The demographic and baseline characteristics are summarized in Table 1. Male subjects accounted for the majority in both cohorts, representing 67.5% and 80.6% of the fasting and fed cohorts, respectively. In the fasting cohort, the mean age, height, body weight, and BMI were 33.53 ± 10.00 years, 169.19 ± 7.86 cm, 63.98 ± 8.67 kg, and 22.30 ± 2.19 kg/m^2^, respectively. The corresponding values in the fed cohort were 31.94 ± 9.93 years, 170.24 ± 7.59 cm, 62.67 ± 8.48 kg, and 21.56 ± 1.92 kg/m^2^.

**TABLE 1:** Demographic and baseline characteristics.

Characteristic	Fasting (n = 40)	Fed (n = 36)
Age (years)		
Mean ± SD	33.53 ± 10.00	31.94 ± 9.93
Min–max	18.00 – 52.00	18.00 – 53.00
Gender, n (%)		
Male	27 (67.5)	29 (80.6)
Female	13 (32.5)	7 (19.4)
Height (cm)		
Mean ± SD	169.19 ± 7.86	170.24 ± 7.59
Min–max	151.50 – 188.00	155.00 – 183.00
Weight (kg)		
Mean ± SD	63.98 ± 8.67	62.67 ± 8.48
Min–max	49.00 – 84.50	48.00 – 83.00
BMI (kg/m²)		
Mean ± SD	22.30 ± 2.19	21.56 ± 1.92
Min–max	19.30 – 26.70	19.20 – 26.50

BMI, body mass index; SD, standard deviation.

### 3.2 Pharmacokinetics and bioequivalence

In the fasting cohort, all 40 subjects were included in the pharmacokinetic concentration set (PKCS). For the pharmacokinetic parameter set (PKPS), 39 subjects provided evaluable data for both the test and reference products. This was because the two subjects who withdrew prior to Period two dosing still provided valid single-period pharmacokinetic data, while the subject who withdrew after Period two dosing had already completed the required pharmacokinetic sampling. In the fed cohort, 34 subjects were included in both the PKCS and the PKPS after excluding the subject who failed to receive the study drug and the subject who withdrew due to an AE. Table 2 summarizes the pharmacokinetic parameters following administration of the test and reference products. Overall, the pharmacokinetic parameters of sitagliptin and metformin were comparable between the two formulations under both fasting and fed conditions. The mean plasma concentration–time profiles (linear and semi-logarithmic scales) for both drugs demonstrated similar trends during the treatment periods (Figure 2).

**TABLE 2:** Summary of pharmacokinetic parameters of sitagliptin and metformin following administration of the test and reference formulations in healthy Chinese subjects.

PK parameters	Sitagliptin - Test (n = 39/n = 34)	Sitagliptin - Reference (n = 39/n = 34)	Metformin - Test (n = 39/n = 34)	Metformin - Reference (n = 39/n = 34)
Under fasting conditions, arithmetic mean ± SD (CV%)				
T_max_^*^ (h)	2.33 (0.67,5.00)	2.33 (0.67,6.00)	3.50 (2.02,8.00)	3.50 (2.33,6.01)
C_max_ (ng/mL)	206.64 ± 69.06 (33.42)	217.55 ± 66.96 (30.78)	959.31 ± 241.63 (25.19)	966.59 ± 222.76 (23.05)
AUC_0-t_ (ng·h/mL)	1736.82 ± 291.95 (16.81)	1714.41 ± 327.21 (19.09)	7329.65 ± 1815.29 (24.77)	7186.48 ± 1627.54 (22.65)
${\text{AUC}}_{0–\infty }$ (ng·h/mL)	1778.17 ± 298.28 (16.77)	1768.56 ± 305.42 (17.27)	7427.08 ± 1806.14 (24.32)	7353.28 ± 1548.10 (21.05)
λ_z_ (h^-1^)	0.08 ± 0.01 (15.91)	0.08 ± 0.02 (19.87)	0.15 ± 0.05 (30.80)	0.15 ± 0.05 (30.79)
t_1/2_ (h)	9.24 ± 1.63 (17.60)	8.82 ± 1.59 (18.01)	5.10 ± 1.33 (26.13)	6.11 ± 7.74 (126.79)
AUC__%Extrap_ (%)	2.33 ± 0.96 (41.36)	3.23 ± 6.61 (204.68)	1.46 ± 0.85 (58.09)	2.52 ± 5.46 (217.10)
Under fed conditions, arithmetic mean ± SD (CV%)				
T_max_^*^ (h)	1.67 (0.67,4.50)	1.67 (1.00,4.50)	6.00 (4.00,10.00)	6.51 (4.00,8.00)
C_max_ (ng/mL)	207.41 ± 54.45 (26.25)	218.41 ± 43.08 (19.72)	613.29 ± 129.41 (21.10)	637.18 ± 107.47 (16.87)
AUC_0-t_ (ng·h/mL)	1619.85 ± 300.94 (18.58)	1638.75 ± 280.03 (17.09)	7203.32 ± 1660.98 (23.06)	7478.75 ± 1369.88 (18.32)
${\text{AUC}}_{0–\infty }$ (ng·h/mL)	1672.24 ± 312.72 (18.70)	1691.10 ± 283.89 (16.79)	7296.33 ± 1664.59 (22.81)	7601.28 ± 1315.69 (17.31)
λ_z_ (h^-1^)	0.07 ± 0.01 (20.12)	0.07 ± 0.01 (17.53)	0.14 ± 0.04 (27.39)	0.14 ± 0.04 (32.20)
t_1/2_ (h)	10.17 ± 2.62 (25.72)	10.37 ± 1.87 (18.00)	5.57 ± 3.29 (59.00)	6.80 ± 7.16 (105.29)
AUC__%Extrap_ (%)	3.07 ± 1.80 (58.60)	3.13 ± 1.27 (40.71)	1.32 ± 0.65 (49.45)	1.82 ± 3.11 (171.20)

**FIGURE 2:**
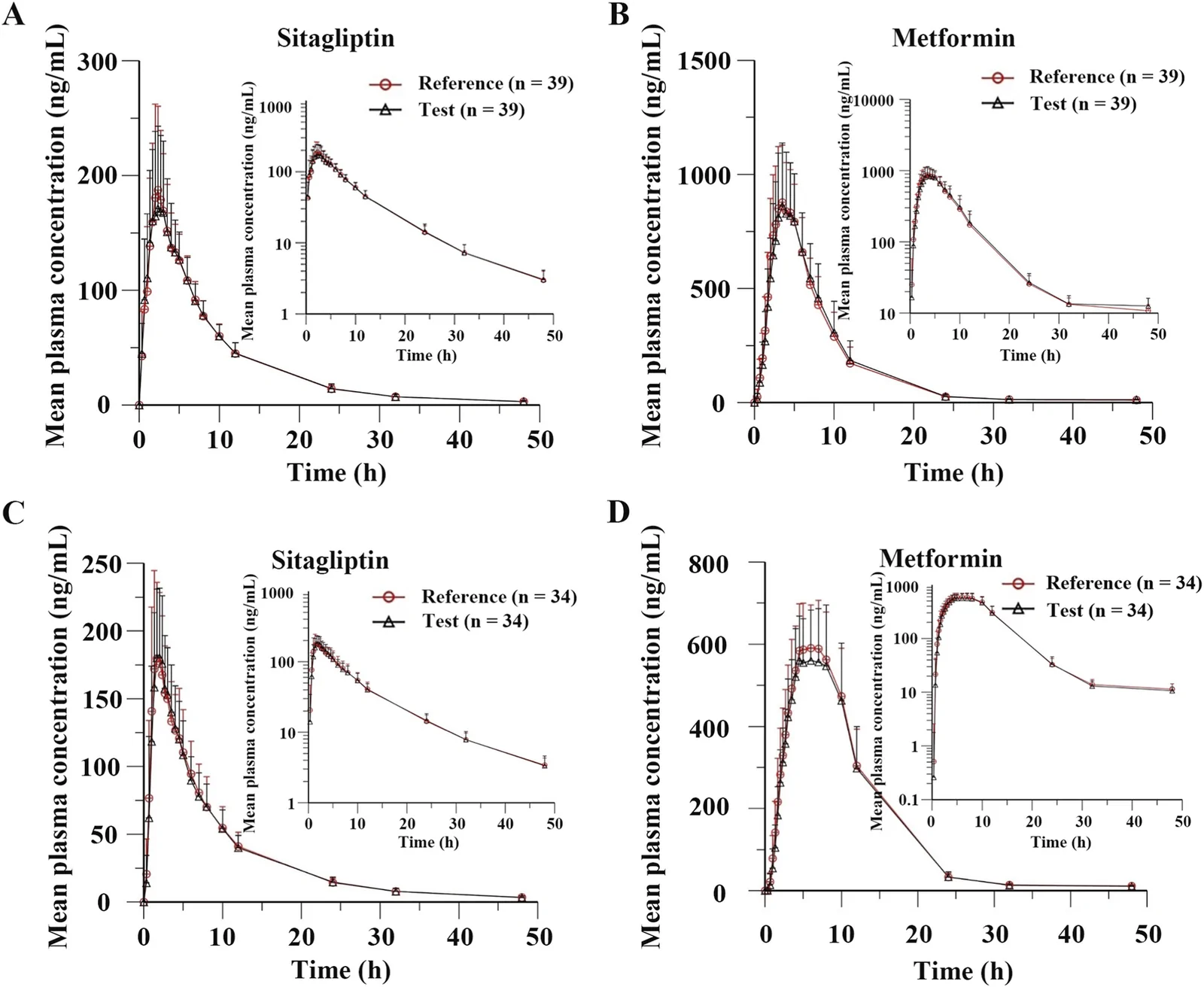
Mean plasma concentration–time profiles of sitagliptin and metformin following administration of the test and reference formulations in healthy Chinese subjects under fasting and fed conditions. Arithmetic mean plasma concentration–time curves of sitagliptin under fasting **(A)** and fed **(C)** conditions, and metformin under fasting **(B)** and fed **(D)** conditions. The main panels represent a linear scale, and the insets represent a semi-logarithmic scale. Error bars denote the standard deviation (SD). T, test formulation; R, reference formulation.

Under fasting conditions, the GMRs (test/reference) and 90% CIs of sitagliptin for C_max_, AUC_0–t_, and ${\text{AUC}}_{0–\infty }$ were 94.62% (88.24%–101.46%), 100.66% (98.24%–103.15%), and 100.84% (98.41%–103.33%), respectively. Under fed conditions, the corresponding values were 93.52% (88.29%–99.06%), 98.58% (96.19%–101.02%), and 98.53% (96.07%–101.04%), respectively. All 90% CIs fell within the predefined bioequivalence range of 80.00%–125.00% (Table 3). As shown in Table 4, the 90% CIs for the metformin pharmacokinetic parameters also met the bioequivalence criteria under both fasting and fed conditions (fasting: C_max_: 93.23%–103.26%, AUC_0–t_: 93.00%–106.20%, ${\text{AUC}}_{0–\infty }$ : 92.89%–105.74%; fed: C_max_: 92.06%–99.39%, AUC_0–t_: 91.31%–100.06%, ${\text{AUC}}_{0–\infty }$ : 91.00%–99.30%).

**TABLE 3:** Bioequivalence assessment of sitagliptin based on Cmax, AUC_0-t_, and ${\text{AUC}}_{0–\infty }$ following administration of the test and reference formulations in healthy Chinese subjects under fasting and fed conditions.

PK parameter	n (Test)	n (Reference)	GM (test)	GM (Reference)	GMR (T/R, %)	CVwR (%)	90% CI (%)
Under fasting condition							
C_max_ (ng/mL)	39	39	196.31	207.48	94.62	18.26	88.24, 101.46
AUC_0-t_ (ng·h/mL)	39	38	1714.77	1703.45	100.66	6.24	98.24, 103.15
${\text{AUC}}_{0–\infty }$ (ng·h/mL)	39	38	1756.04	1741.39	100.84	6.22	98.41, 103.33
Under fed conditions							
C_max_ (ng/mL)	34	34	200.45	214.34	93.52	14.07	88.29, 99.06
AUC_0-t_ (ng·h/mL)	34	34	1592.38	1615.40	98.58	5.96	96.19, 101.02
${\text{AUC}}_{0–\infty }$ (ng·h/mL)	34	34	1643.11	1667.68	98.53	6.15	96.07, 101.04

GM, geometric mean; GMR, geometric mean ratio; CVwR, within-subject coefficient of variation of the reference formulation; CI, confidence interval.

**TABLE 4:** Bioequivalence assessment of metformin based on Cmax, AUC_0–t_, and ${\text{AUC}}_{0–\infty }$ following administration of the test and reference formulations in healthy Chinese subjects under fasting and fed conditions.

PK parameter	n (Test)	n (Reference)	GM (Test)	GM (Reference)	GMR (T/R, %)	CVwR (%)	90% CI (%)
Under fasting conditions							
C_max_ (ng/mL)	39	39	921.72	939.40	98.12	13.30	93.23, 103.26
AUC_0-t_ (ng·h/mL)	39	38	7042.76	7086.36	99.38	17.17	93.00, 106.20
${\text{AUC}}_{0–\infty }$ (ng·h/mL)	39	38	7147.22	7211.40	99.11	16.76	92.89, 105.74
Under fed conditions							
C_max_ (ng/mL)	34	34	600.84	628.17	95.65	9.34	92.06, 99.39
AUC_0-t_ (ng·h/mL)	34	34	7023.60	7347.89	95.59	11.17	91.31, 100.06
${\text{AUC}}_{0–\infty }$ (ng·h/mL)	34	34	7117.71	7487.91	95.06	10.65	91.00, 99.30

GM, geometric mean; GMR, geometric mean ratio; CVwR, within-subject coefficient of variation of the reference formulation; CI, confidence interval.

ANOVA results indicated no significant effects of sequence, period, or formulation on the ln-transformed pharmacokinetic parameters of sitagliptin under either fasting or fed conditions (all *P* > 0.05; Supplementary Table S1). For metformin, a significant period effect was observed for AUC_0–t_ and ${\text{AUC}}_{0–\infty }$ under fasting conditions (*P* < 0.05), whereas sequence and formulation effects were not significant. No significant sequence, period, or formulation effects were observed under fed conditions (Supplementary Table S2).

### 3.3 Safety

Safety evaluations were based on the safety analysis set (SS). In the fasting cohort (SS = 40), 12 AEs occurred in eight subjects (20.0%). These included 9 Grade 1 events (in eight subjects) and 3 Grade 2 events (in two subjects). One subject discontinued the study due to an AE (elevated blood hCG levels). Following administration of the test product, 5 AEs occurred in four subjects (10.3%, 4/39), while 7 AEs occurred in four subjects (10.3%, 4/39) for the reference product. Treatment-related adverse events (TRAEs) were reported in three subjects (3 events; 7.5%), comprising one event for the test product and two events for the reference product. At the system organ class (SOC) level, Investigations was the most common category of AEs (9 events in six subjects), followed by blood and lymphatic system disorders (2 events in two subjects) and cardiac disorders (1 event in one subject). “Anaemia” and “Blood cholesterol increased” were the most frequently reported PTs, each occurring in 2 subjects (5.0%). each being reported in 2 subjects (5.0%). Two events of urine leukocyte positive were reported in 1 subject (2.5%) (Table 5).

**TABLE 5:** Summary of adverse events in the safety analysis set under fasting and fed conditions.

Category	Under fasting conditions						Under fed conditions					
	Test (n = 39)		Reference (n = 39)		Total (N = 40)		Test (n = 34)		Reference (n = 35)		Total (N = 35)	
​	Events	Subjects n (%)	Events	Subjects n (%)	Events	Subjects n (%)	Events	Subjects n (%)	Events	Subjects n (%)	Events	Subjects n (%)
Summary of AEs												
Overall AEs	5	4 (10.3)	7	4 (10.3)	12	8 (20.0)	4	4 (11.8)	6	5 (14.3)	11	8 (22.9)
Treatment-related AEs	1	1 (2.6)	2	2 (5.1)	3	3 (7.5)	3	3 (8.8)	0	0 (0)	3	3 (8.6)
AEs leading to discontinuation	0	0 (0.0)	1	1 (2.6)	1	1 (2.5)	0	0 (0.0)	1	1 (2.9)	1	1 (2.9)
Investigations												
White blood cell count decreased	0	0 (0.0)	1	1 (2.6)	1	1 (2.5)	0	0 (0.0)	0	0 (0.0)	0	0 (0.0)
Neutrophil count decreased	0	0 (0.0)	1	1 (2.6)	1	1 (2.5)	0	0 (0.0)	0	0 (0.0)	0	0 (0.0)
Blood human chorionic gonadotropin increased	0	0 (0.0)	1	1 (2.6)	1	1 (2.5)	0	0 (0.0)	0	0 (0.0)	0	0 (0.0)
Urine white blood cells positive	1	1 (2.6)	0	0 (0.0)	1	1 (2.5)	0	0 (0.0)	0	0 (0.0)	0	0 (0.0)
Urine white blood cell count increased	1	1 (2.6)	0	0 (0.0)	1	1 (2.5)	0	0 (0.0)	0	0 (0.0)	0	0 (0.0)
Blood triglycerides increased	0	0 (0.0)	1	1 (2.6)	1	1 (2.5)	0	0 (0.0)	0	0 (0.0)	0	0 (0.0)
Alanine aminotransferase increased	0	0 (0.0)	1	1 (2.6)	1	1 (2.5)	0	0 (0.0)	0	0 (0.0)	0	0 (0.0)
Blood cholesterol increased	1	1 (2.6)	1	1 (2.6)	2	2 (5.0)	0	0 (0.0)	0	0 (0.0)	0	0 (0.0)
Blood uric acid increased	0	0 (0.0)	0	0 (0.0)	0	0 (0.0)	1	1 (2.9)	1	1 (2.9)	2	2 (5.7)
Pulse rate increased	0	0 (0.0)	0	0 (0.0)	0	0 (0.0)	0	0 (0.0)	1	1 (2.9)	1	1 (2.9)
Cardiac disorders												
Sinus tachycardia	1	1 (2.6)	0	0 (0.0)	1	1 (2.5)	1	1 (2.9)	2	2 (5.7)	3	3 (8.6)
Blood and lymphatic system disorders												
Anemia	1	1 (2.6)	1	1 (2.6)	2	2 (5.0)	0	0 (0.0)	0	0 (0.0)	0	0 (0.0)
Gastrointestinal disorders												
Nausea	0	0 (0.0)	0	0 (0.0)	0	0 (0.0)	1	1 (2.9)	0	0 (0.0)	1	1 (2.9)
Nervous system disorders												
Epileptic seizure	0	0 (0.0)	0	0 (0.0)	0	0 (0.0)	0	0 (0.0)	1	1 (2.9)	1	1 (2.9)
Infections and infestations												
Rhinitis	0	0 (0.0)	0	0 (0.0)	0	0 (0.0)	1	1 (2.9)	0	0 (0.0)	1	1 (2.9)
Renal and urinary disorders												
Nephrolithiasis	0	0 (0.0)	0	0 (0.0)	0	0 (0.0)	0	0 (0.0)	1	1 (2.9)	1	1 (2.9)

AE, adverse event.

In the fed cohort (SS = 35), 11 AEs were reported in eight subjects (22.9%). These included 9 Grade 1 events (in seven subjects) and 2 Grade 2 events (in two subjects). One subject discontinued the study due to an AE (epileptic seizure). Following administration of the test product, 4 AEs occurred in four subjects (11.8%, 4/34), compared with 6 AEs in five subjects (14.3%, 5/35) for the reference product. Three TRAEs were reported in three subjects, all associated with the test product. No deaths or serious adverse events (SAEs) occurred throughout the study. At the PT level, sinus tachycardia had the highest subject incidence, occurring in 3 subjects (8.6%). including 1 subject following administration of the test product and 2 subjects following administration of the reference product (Table 5).

## 4 Discussion

The present study evaluated the pharmacokinetics, bioequivalence, and safety of a generic sitagliptin/metformin hydrochloride XR tablet (50 mg/500 mg) relative to the reference product, Janumet® XR, in healthy Chinese subjects under fasting and fed conditions. The findings demonstrated that the test and reference formulations exhibited comparable pharmacokinetic characteristics for both sitagliptin and metformin (Migoya et al., 2010; Chen et al., 2016; Que et al., 2023). For all primary pharmacokinetic parameters, namely, Cmax, AUC_0–t_, and ${\text{AUC}}_{0–\infty }$ , the 90% confidence intervals of the geometric mean ratios were entirely contained within the predefined bioequivalence range of 80.00%–125.00%, thereby establishing bioequivalence under both dosing conditions. A noteworthy observation was the statistically significant period effect detected for metformin AUC_0–t_ and ${\text{AUC}}_{0–\infty }$ under fasting conditions. In two-period crossover bioequivalence trials, period effects are not uncommon and may reflect temporal variation between study periods rather than differences attributable to the formulations themselves (Lim and In, 2021; Koehne-Voss et al., 2011). Such variation may arise from nonspecific within-subject changes, including subtle physiological fluctuations or other uncontrolled time-related factors. Importantly, the interpretation of bioequivalence depends primarily on the estimated test/reference ratios and their associated confidence intervals, rather than on the isolated presence of a period effect (Lim and In, 2021; Koehne-Voss et al., 2011). In the present analysis, no significant formulation effect was identified for metformin, and all primary metformin exposure metrics satisfied the predefined bioequivalence criteria. Accordingly, the observed period effect is unlikely to have affected the validity of the overall bioequivalence conclusion.

The results under fed conditions provide additional support for the formulation performance of the generic XR product. Assessment under fed conditions is particularly relevant for extended-release formulations, as food intake may alter gastrointestinal transit, luminal environment, and drug absorption kinetics (Charman et al., 1997; Garbacz and Klein, 2012). In this study, both sitagliptin and metformin remained bioequivalent following administration with a high-fat, high-calorie meal, indicating that the release and absorption characteristics of the test product were comparable to those of the reference formulation in the fed state (Que et al., 2023). Taken together with the fasting results, these findings suggest that the generic formulation has consistent *in vivo* performance across clinically relevant dosing conditions.

Both formulations were generally well tolerated in this study. The incidence, severity, and overall pattern of AEs were broadly similar between the test and reference products under both fasting and fed conditions. Most AEs were mild or moderate in intensity, and no deaths or SAEs were reported. The observed events, which were mainly laboratory abnormalities and isolated clinical findings such as sinus tachycardia, were consistent with the safety observations typically encountered in phase I bioequivalence studies and did not indicate any new or unexpected safety concerns (Migoya et al., 2010; Chen et al., 2016; Que et al., 2023). Overall, the safety findings support the short-term tolerability of the generic formulation in healthy subjects.

Several limitations should be acknowledged. First, the study was conducted in healthy volunteers rather than in patients with T2DM, and disease-related factors may influence the pharmacokinetic behavior of metformin, and potentially the combination product overall, in clinical practice (Duong et al., 2013). Second, this was a single-dose study; although this design is appropriate and standard for regulatory bioequivalence assessment, it does not provide information on steady-state pharmacokinetics or long-term tolerability. Third, the sample size was determined to demonstrate bioequivalence and was not intended to detect rare AEs or small differences in safety outcomes. Nevertheless, these limitations are inherent to most conventional bioequivalence studies and do not compromise the primary objective of establishing formulation equivalence.

In conclusion, the generic sitagliptin/metformin hydrochloride XR tablet (50 mg/500 mg) was bioequivalent to Janumet® XR under both fasting and fed conditions in healthy Chinese subjects, and both products were generally well tolerated. These findings support the generic formulation as a therapeutically equivalent alternative to the reference product and provide evidence for its potential clinical use in the management of T2DM.

## Data Availability

The raw data supporting the conclusions of this article will be made available by the authors, without undue reservation.
