# Supplementary material for: Bioequivalence study of two formulations of a sitagliptin/metformin hydrochloride extended-release tablet in healthy Chinese subjects under fasting and fed conditions: a randomized, open-label, single-dose, crossover trial
Source: Front Pharmacol. 2026 Oct 14;17:1927547. doi: 10.3389/fphar.2026.1927547 (PMC13713397; doi:10.3389/fphar.2026.1927547)
Supplement: Supplementary file 1 [file DataSheet1.pdf]

Supplementary Materials

Supplementary Appendix S1. Detailed Inclusion and Exclusion Criteria

Inclusion criteria

Participants were eligible for enrollment only if they met all of the following criteria:

1. Voluntarily signed the informed consent form before any study-related procedures and were fully informed of the study objectives, procedures, and possible adverse reactions.
2. Were able and willing to comply with all study requirements according to the protocol.
3. Agreed to have no plans for pregnancy, sperm donation, or oocyte donation from the time of signing informed consent until 3 months after the last dose. For female participants, this requirement applied from 14 days before signing informed consent until 3 months after the last dose. All participants agreed to use effective contraception during the study period, with non-pharmacological contraception required during the trial.
4. Healthy male or female adults aged 18 years or older (including 18 years).
5. Body weight of at least 50 kg for males and 45 kg for females, with a body mass index (BMI) of 19.0–27.0 kg/m^2 (inclusive).

Exclusion criteria

Participants were excluded if they met any of the following criteria:

1. History of specific allergic conditions (eg, urticaria, exfoliative dermatitis, angioedema, or bronchial hyperreactivity), allergic constitution (eg, allergy to two or more drugs, foods such as milk, or pollen), or known hypersensitivity to any component of the study drug or related compounds.
2. History of clinically significant disease involving the cardiovascular, hepatic, renal, gastrointestinal, neurological, endocrine, respiratory, or psychiatric systems, or any other condition that, in the investigator’ s judgment, made the participant unsuitable for the study.

3. Any clinically significant abnormality identified by the investigator during screening, including abnormalities in physical examination, vital signs, 12-lead electrocardiogram, or clinical laboratory tests.

4. Previous or current history of pancreatitis.

5. Previous history of hypoglycemia.

6. Estimated glomerular filtration rate (eGFR) <90 mL/min/1.73 m^2, calculated using the simplified MDRD equation:

$GFR = 186 \times SCr^{-1.154} \times age^{-0.203} \times (0.742 \text{ for females}) \times 1.233$.

7. History of dysphagia or any gastrointestinal disease that could affect drug absorption, such as gastrectomy, small bowel resection, atrophic gastritis, gastrointestinal bleeding, or gastrointestinal obstruction.

8. Difficulty with blood collection, intolerance to venipuncture, or history of syncope related to blood drawing or needle insertion.

9. Females who were pregnant, lactating, or had a positive pregnancy test result.

10. Positive test result for any of the following: hepatitis B surface antigen, hepatitis C antibody, human immunodeficiency virus antibody, or syphilis antibody.

11. History of drug abuse within the previous 5 years, use of illicit drugs within 3 months before screening, or a positive urine drug screening result.

12. Average alcohol consumption of more than 14 units/week within 3 months before screening (1 unit = 360 mL beer, 45 mL spirits containing 40% alcohol, or 150 mL wine), unwillingness to abstain from alcohol from 48 hours before dosing until completion of blood sampling in each period, or a positive alcohol breath test.

13. Average smoking of more than 5 cigarettes per day within 3 months before screening, or unwillingness to abstain from all tobacco products during confinement.

14. History of surgery within 3 months before first dosing, or planned surgery during the study period.

15. Blood donation or significant blood loss (>400 mL, excluding physiological blood loss in women) within 3 months before first dosing, or platelet donation of 2 therapeutic units within 1 month before first dosing (1 therapeutic unit = 12 U platelets).

16. Participation in any drug clinical trial and receipt of study medication within 3 months before first dosing.
17. Use of any prescription medication within 14 days before dosing.
18. Use of any over-the-counter medication, herbal medicine, or health supplement within 7 days before dosing.
19. Consumption of special foods or beverages (eg, grapefruit or grapefruit-containing products), engagement in strenuous exercise, or presence of any other factor that might affect drug absorption, distribution, metabolism, or excretion within 48 hours before dosing.
20. Consumption of foods or beverages rich in xanthines (eg, coffee, tea, chocolate, cocoa, or milk tea) within 48 hours before dosing.
21. Special dietary requirements or inability to comply with the standardized meals and study-site dietary restrictions.
22. Occurrence of any acute illness during the screening period or before administration of study medication.
23. Any other condition that, in the opinion of the investigator, made the participant unsuitable for participation in the study.

Supplementary Table S1. ANOVA results for ln-transformed sitagliptin Cmax, AUC_{0-t}, and $AUC_{0-\infty}$ under fasting and fed conditions.

**Effect**	**ln(C_{max})**	**ln(AUC_{0-t})**	**ln($AUC_{0-\infty}$)**
Under fasting conditions			
Sequence	0.5548	0.5697	0.3951
Formulation	0.1891	0.3024	0.4088
Period	0.5069	0.7966	0.1170
Under fed conditions			
Sequence	0.8483	0.5362	0.5640
Formulation	0.0573	0.3276	0.3263
Period	0.9974	0.4489	0.3283

Supplementary Table S2. ANOVA results for ln-transformed metformin Cmax, AUC_{0-t}, and $AUC_{0-\infty}$ under fasting and fed conditions.

**Effect**	**ln(C_{max})**	**ln(AUC_{0-t})**	**ln($AUC_{0-\infty}$)**
Under fasting conditions			
Sequence	0.3075	0.5093	0.4697
Formulation	0.5345	0.8762	0.8172
Period	0.1947	0.0384	0.0459
Under fed conditions			
Sequence	0.1440	0.2751	0.3739
Formulation	0.0838	0.1217	0.0577
Period	0.0676	0.3822	0.4805
